# Signaling Pathways Driving MSC Osteogenesis: Mechanisms, Regulation, and Translational Applications

**DOI:** 10.3390/ijms26031311

**Published:** 2025-02-04

**Authors:** Liuqing Wang, Minjie Ruan, Qiqi Bu, Chengzhu Zhao

**Affiliations:** Laboratory of Skeletal Development and Regeneration, Key Laboratory of Clinical Laboratory Diagnostics (Ministry of Education), College of Laboratory Medicine, Chongqing Medical University, Chongqing 400016, China

**Keywords:** mesenchymal stem cells (MSCs), osteogenesis, bone diseases, bone regeneration, bone tissue engineering

## Abstract

Mesenchymal stem cells (MSCs) are crucial for skeletal development, homeostasis, and repair, primarily through their differentiation into osteoblasts and other skeletal lineage cells. Key signaling pathways, including Wnt, TGF-β/BMP, PTH, Hedgehog, and IGF, act as critical regulators of MSC osteogenesis, playing pivotal roles in maintaining bone homeostasis and facilitating regeneration. These pathways interact in distinct ways at various stages of bone development, mineralization, and remodeling. This review provides an overview of the molecular mechanisms by which these pathways regulate MSC osteogenesis, their influence on bone tissue formation, and their implications in bone diseases and therapeutic strategies. Additionally, we explore the potential applications of these pathways in bone tissue engineering, with a particular focus on promoting the use of MSCs as seed cells for bone defect repair. Ultimately, this review aims to highlight potential avenues for advancing bone biology research, treating bone disorders, and enhancing regenerative medicine.

## 1. Introduction

The skeletal system is a vital tissue in the human body, serving multiple functions such as support, protection, movement, and mineral storage. Its development and maintenance depend on complex biological processes that involve the precise regulation of various signaling pathways, which control bone development, formation, and remodeling by influencing the proliferation, differentiation, and activity of various bone-resident cells. Over the years, research has revealed the central role of several key signaling pathways, with the most important being the wingless/integrated (Wnt), transforming growth factor-beta (TGF-β)/bone morphogenetic proteins (BMP), parathyroid hormone (PTH), Hedgehog (Hh), and insulin-like growth factor (IGF) signaling pathways. The Wnt signaling pathway is considered a critical regulator of bone formation, primarily promoting osteogenesis by regulating the differentiation of mesenchymal stem cells (MSCs). The TGF-β/BMP signaling axis plays a key role in bone development, particularly in bone formation and repair, by regulating the proliferation and differentiation of osteoprogenitor cells, thereby promoting the production of the bone matrix. The PTH signaling pathway regulates the balance between osteoblasts and osteoclasts, ensuring the stability of bone metabolism and playing an important role in bone mass regulation. While the role of the Hh signaling pathway in bone is relatively less studied, it has a significant impact on cartilage formation, endochondral ossification, and bone growth. The IGF signaling pathway is crucial for skeletal growth, osteoblast proliferation, differentiation, and survival [1,2,3,4]. These signaling pathways interact and cooperate to maintain skeletal homeostasis.

**Figure 1:**
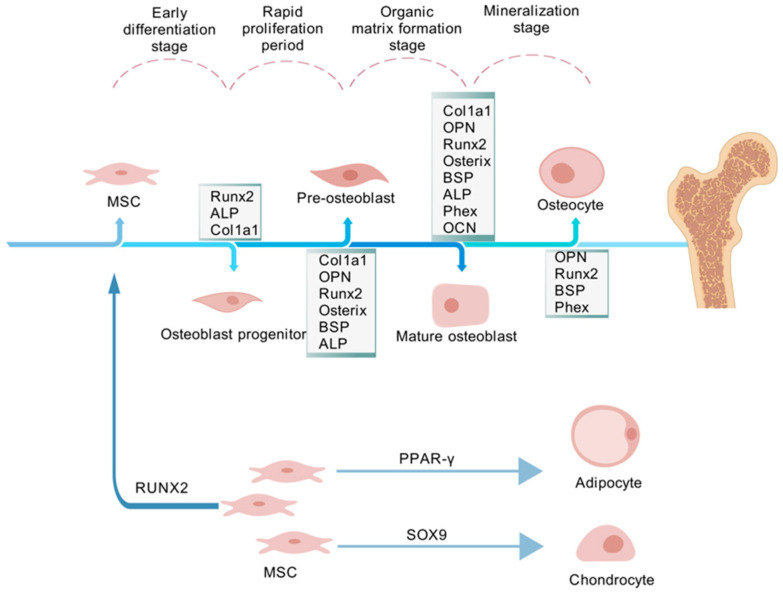
The osteogenic differentiation process of MSCs. Runx2 = runt-related transcription factor 2; ALP = alkaline phosphatase; Col1a1 = collagen type I alpha 1; OPN = osteopontin; BSP = bone sialoprotein; Phex = phosphate-regulating endopeptidase homolog; OCN = osteocalcin; PPAR-γ = peroxisome proliferator-activated receptor gamma; SOX9 = SRY-box transcription factor 9. The figures are created with BioGDP.com.

MSCs originate from embryonic mesoderm and neural crest and can be isolated from various tissues, including adipose tissue, bone marrow, placenta, and umbilical cord blood [5]. Possessing significant differentiation potential, MSCs can become osteoblasts, chondrocytes, adipocytes, myocytes, neurons, or keratinocytes, regulated by transcription factors influenced by growth factors and their microenvironment [6,7,8,9]. For instance, osteogenic differentiation is driven by the genes runt-related transcription factor 2 (Runx2), distal-less homeobox 5 (Dlx5), and osterix (Osx). Under the influence of PPAR-γ, MSCs differentiate into adipocytes, while SOX9 promotes differentiation into the chondrocyte lineage. Osteogenic differentiation of MSCs is a programmed process, involving several stages and distinct cell types. In the early differentiation stage, osteoblast progenitors express markers such as alkaline phosphatase (ALP), Runx2, and collagen type I alpha 1 (Col1a1). Following rapid proliferation, the expression of ALP and Runx2 increases, and osteopontin (OPN), Osterix, and bone sialoprotein (BSP) are detected in pre-osteoblasts. During the organic matrix formation stage, the expression of various markers becomes more pronounced. In mature osteoblasts, the expression of Runx2, ALP, OPN, Osterix, BSP, Col1a1, OCN, and the phosphate-regulating gene with homologies to endopeptidases on the X chromosome (Phex) is elevated. At the mineralization stage, mature osteoblasts gradually transform into osteocytes, with markers such as Phex, OPN, Runx2, and BSP detectable (Figure 1) [10]. However, the precise roles of key signaling pathways in MSC osteogenesis are not yet fully understood. A deeper understanding of these pathways will enhance insights into bone biology and contribute to the creation of innovative bone disease treatments.

Furthermore, MSCs, as functional seed cells, are widely applied in bone tissue engineering. In orthopedics, large bone defects caused by pathological diseases or trauma pose significant challenges, with the shortage of donor bone tissue further limiting the effectiveness of clinical treatments [11]. While artificial bone grafts offer a solution to the scarcity of donor tissue, their insufficient biological activity can hinder effective bone regeneration. MSCs, with their robust proliferative capacity, high biological activity, and minimal immunogenicity, have emerged as invaluable tools for enhancing the biological performance of artificial bone grafts [7]. In bone tissue engineering, identifying suitable strategies to promote MSC osteogenic differentiation is pivotal for promoting graft integration and supporting defect regeneration. To achieve this, innovative approaches are being developed to functionalize scaffolds by targeting key signaling molecules. These strategies involve integrating critical signaling molecules, their upstream or downstream regulators, natural compounds, or biological polymers into advanced scaffold materials such as electrospun nanofibers, bioceramics, and 3D-printed polycaprolactone/polylactic acid composites [12,13,14]. By further investigating the biological properties of MSCs and their differentiation processes under the regulation of different signaling pathways, more precise treatment strategies for bone repair and regeneration can be developed.

In this context, this review will focus on how five key signaling pathways—Wnt; TGF-β/BMP; PTH; Hh; and IGF—cooperate to regulate bone system development; homeostasis; and regeneration; as well as their clinical applications in bone disease treatment. A deeper understanding of these signaling pathways and their interactions could aid in advancing fundamental research and the development of novel therapeutic approaches.

## 2. Wnt Signaling Pathway

### 2.1. Overview of Wnt Signaling

The Wnt signaling pathway is mediated by a family of glycoproteins, playing a significant role in the proliferation and differentiation of MSCs. Wnt ligands are universally expressed across all animal species. In mammals, 19 distinct Wnt ligands contribute to the complexity of the Wnt signaling cascade. These cysteine-rich proteins contain an N-terminal signal peptide for secretion and require lipid modifications to become biologically active [15].

The Wnt signaling pathway is divided into canonical (β-catenin-dependent) and non-canonical (β-catenin-independent) pathways. In the canonical pathway, Wnt ligands (e.g., Wnt1, Wnt3a, Wnt10) bind to Frizzled (Fzd) receptors and low-density lipoprotein receptor-related protein (LRP) 5/6, initiating downstream signaling. Co-receptors like ROR1 and Ryk also interact with Fzd to modulate signal transduction [7,10,11]. Ligand-receptor binding leads to the formation of a complex with LRP5/6 and Dishevelled (Dvl), which recruits components of the destruction complex (GSK3β and APC) to the cell membrane, inhibiting β-catenin degradation. This stabilizes β-catenin, allowing its accumulation and translocation to the nucleus, where it activates target genes like Axin2 and Runx2, which in turn activate Wnt-mediated processes such as osteogenesis [16].

The non-canonical Wnt pathway operates independently of β-catenin. Ligands like Wnt5a, Wnt7, and Wnt11 activate small GTPases (Rho and Rac) through Fzd receptors, triggering the JNK cascade via Dvl proteins to regulate cytoskeletal reorganization and cell polarity. In the Wnt/Ca^2+^ pathway, ligands activate the Fzd receptor, triggering Gα_11_-mediated activation of phospholipase Cβ (PLCβ). PLCβ hydrolyzes PIP_2_ to produce IP_3_ and DAG, leading to intracellular Ca^2+^ release. This activates calmodulin, Ca^2+^/calmodulin-dependent protein kinase II (CaMKII), protein kinase C (PKC), and ultimately Nuclear Factor of Activated T-cells (NFAT) transcription factors. Notably, non-canonical pathways can antagonize canonical Wnt/β-catenin signaling, maintaining cellular balance [17,18,19].

### 2.2. The Role of Wnt Pathway Regulation in MSC Osteogenic Differentiation

The Wnt signaling pathway is important in regulating skeletal development and homeostasis. LRP5/6 are single-pass transmembrane receptors, belonging to the low-density lipoprotein receptor family [20]. Heterozygous mutations in LRP5 are strongly associated with high bone mass syndrome, as they lead to increased bone formation markers like OCN and ALP, enhancing bone density by stimulating osteoblast differentiation [21]. The TGF-β signaling pathway upregulates LRP5 expression, thereby facilitating the activation of the Wnt/β-catenin pathway [22]. Regulatory proteins such as sclerostin (SOST), Wise, and Dickkopf (DKK) can competitively bind to LRP5/6 and inhibit the Wnt signaling [23,24]. SOST, primarily secreted by osteocytes, negatively regulates bone formation, affecting bone density and remodeling. Reducing SOST production is essential for activating Wnt target genes and promoting mechanosensitive bone formation. Conversely, SOST overexpression significantly contributes to osteoporosis and other bone density disorders [25]. Neutralizing antibodies against SOST have emerged for treating bone metabolic diseases. Currently, three neutralizing monoclonal SOST antibodies are in clinical trials: two humanized antibodies (Blosozumab by Eli Lilly and Romosozumab by Amgen/UCB) and one fully human antibody (Setrusumab/BPS804 by Novartis), which is derived entirely from the human immune system, resulting in reduced immunogenicity. Romosozumab is the most extensively studied, having completed three Phase III randomized controlled trials (RCTs) in postmenopausal women. In contrast, Blosozumab did not advance beyond Phase II trials, while BPS804 has demonstrated efficacy in Phase I and II trials involving patients with osteogenesis imperfecta and adult hypophosphatasia (Table 1) [24,26].

β-catenin, a core protein in the classical Wnt signaling pathway, is a key target for activation. Both Z-DNA binding protein 1 (ZBP1), an immune signaling protein, and Chrysosplenetin, an active D-methylated flavonoid, enhance β-catenin nuclear translocation in BMSCs, which in turn upregulates osteogenic markers such as Runx2, Bglap, Ctnnb1, and Bmp2, while increasing ALP activity and ECM mineralization to promote osteogenic differentiation [18,32,33]. Targeting β-catenin stability suppressors, such as Axin and GSK3β, has a similar effect. The GSK3β inhibitor lithium chloride increases β-catenin stability and induces its nuclear translocation. In LRP5-deficient mice, lithium chloride restores bone volume to wild-type levels by increasing osteoblast numbers and reversing low bone mass. However, lithium chloride and other GSK3 inhibitors have not been further developed for osteoporosis treatment due to their lack of bone specificity and potential off-target effects [24,34]. Sirtuin 1 (SIRT1), a NAD+-dependent deacetylase, binds to β-catenin and promotes its phosphorylation. Highly expressed in MSCs, osteoblasts, and osteoclasts, SIRT1 plays a key role in bone homeostasis. It enhances MSC viability and osteogenic differentiation under stress, making it a potential target for bone diseases like osteoporosis and osteonecrosis. However, its clinical application is limited by Phase III trial requirements and interactions with non-skeletal tissues [24]. Notch signaling interacts with Wnt signaling to promote osteoprogenitor cell proliferation while inhibiting osteogenic differentiation, likely through downstream effectors such as HES and HEY proteins. These proteins suppress Runx2 expression, suggesting a synergistic role in Wnt-induced osteogenesis [35,36,37].

In the non-canonical Wnt signaling pathway, Wnt7b secreted by osteoblasts promotes MSC osteogenic differentiation through the Wnt-Gαq/11-PKCδ cascade. In mice, reduced expression of Wnt7b and PKCδ leads to decreased embryonic skeletal formation [38]. Wnt5a binds to the co-receptors receptor tyrosine kinase-like orphan receptor 2 (Ror2) and Fzd, activating the Jnk/NK pathway to induce RUNX2 expression while inhibiting PPARγ, thereby promoting bone formation [39]. Under inflammatory conditions, human MSCs are more likely to differentiate into osteoblasts via the non-canonical Wnt pathway, potentially influenced by IL-1β, which induces Ror2 expression and activates MSC osteogenic differentiation [40,41].

### 2.3. Wnt Signaling in MSC Osteogenesis for Bone Regeneration

**Figure 2:**
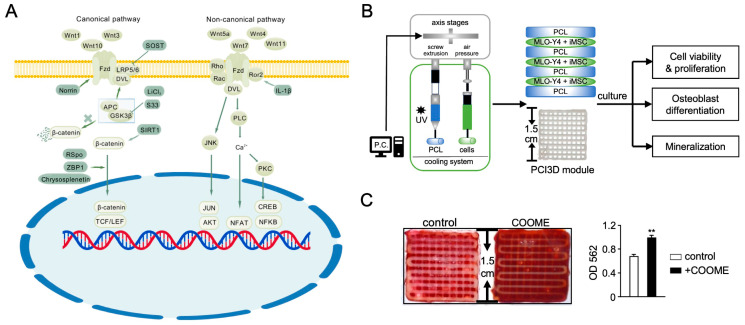
Wnt Signaling in MSC osteogenesis. (**A**) Canonical and non-canonical Wnt signaling pathways regulating MSC osteogenesis. Fzd = Frizzled; LPR = low-density lipoprotein receptor-related protein; DVL = Dishevelled; SOST = sclerostin; APC = adenomatous polyposis coli; GSK3 = glycogen synthase kinase 3; TCF/LEF = T-cell factor/lymphoid enhancer factor; SIRT1 = Sirtuin 1; S33 = SB216763; RSpo = R-spondin; Ror2 = receptor tyrosine kinase-like orphan receptor 2; PLC = phospholipase C; JNK = c-Jun N-terminal kinase; PKC = protein kinase C; AKT = protein kinase B; NFAT = nuclear factor of activated T-cells; CREB = cAMP response element-binding protein; NFκB = nuclear factor kappa-B; IL-1 β = interleukin-1 beta. The figures are created with BioGDP.com. (**B**) Schematic representation of the PCL and cell-integrated 3D (PCI3D) printer, which includes a print module with PCL beams and cell-laden hydrogel beams. (**C**) Wnt-activated osteocytes create an osteogenic microenvironment. CHIR99021 activates Wnt signaling in osteocytes, establishing an osteogenic microenvironment (COOME) that enhances the osteogenic potential of human iPSC-derived MSCs (iMSCs) within the PCI3D system. ** *p* < 0.01 versus the control group using the *t*-test. Reproduced with permission from Guo Q et al. [46].

Targeting key regulators of the Wnt signaling pathway is a promising approach to enhance the efficiency of regenerative therapy. Studies have shown that Apelin-13, a ligand of the G protein-coupled receptor, promotes osteogenic differentiation of human BMSCs via the Wnt/β-catenin pathway in vitro and accelerates bone healing in a rat tibial defect model [42]. Icariin (ICA), an herbal compound, promotes osteogenesis in BMSCs via the Wnt/β-catenin pathway and effectively repairs osteoporotic bone defects in rats when combined with a porous magnesium alloy scaffold [43]. Activation of Wnt signaling in mature osteocytes creates a microenvironment that promotes MSC osteogenesis. The GSK3β inhibitor SB216763 (S33) activates the canonical Wnt pathway in the MLO-Y4 murine osteocyte-like cell line. This activation increases ALP activity and the expression of osteoblast marker genes (including Alpl, Col1α1, and Runx2) in MSCs co-cultured with MLO-Y4, enhancing MSC proliferation, osteogenic differentiation, and mineralization in the co-culture system [44]. The decellularized matrix derived from Wnt-activated osteocytes, when incorporated into a 3D-printed polycaprolactone (PCL) scaffold, demonstrates excellent biocompatibility and supports bone regeneration in critical-sized calvarial defects in mice. This microenvironment supports regeneration and exhibits biological activity, as evidenced by the increased presence of osteoblasts, osteoclasts, H-type blood vessels, and neuro-like cells in the regenerated area [45]. CHIR99021 activates Wnt signaling to create an osteogenic microenvironment in MLOY4, enhancing the osteogenic potential and biological activity of co-cultured human-induced pluripotent stem cell-derived MSCs (iMSCs). Bio3D modules printed with iMSCs and microenvironment components as materials, using a PCL-based 3D cell-integrated system (PCI3D), demonstrate superior osteogenic differentiation and mineralization [46]. The osteogenic microenvironment generated by SKL2001, another Wnt activator, displayed corresponding safety and osteogenesis-promoting characteristics on a 3D scaffold. The PCL-cell integrated modules can stably support cell growth and osteogenic differentiation for over 28 days [47]. These findings highlight the promising potential of Wnt-activated osteocyte-derived microenvironments as a strategy for generating high-quality MSCs in bone tissue engineering (Figure 2).

## 3. TGF-β/BMP Signaling Pathway

### 3.1. Overview of TGF-β/BMP Signaling

TGF-βs and BMPs, both members of the TGF-β superfamily, are critical regulators of osteoblast and chondrocyte differentiation [48]. TGF-β ligands are initially synthesized as precursor peptides, and their activity requires exposure to receptor-binding sites for interaction with receptors. TGF-β activation primarily occurs through integrin-dependent pathways, though integrin-independent mechanisms, such as those mediated by acid, base, reactive oxygen species (ROS), thrombospondin-1 (TSP-1), and proteases, also exist. The mature TGF-β ligand binds to type II receptors (e.g., TGF-β receptor type II), which then recruits type I receptors (e.g., TGF-β receptor type I/activin receptor-like kinase (ALK) 5), forming a tetrameric complex that initiates signaling. TGF-β signaling is primarily transmitted via the canonical SMAD-dependent pathway. Additionally, non-canonical pathways, such as TAK1-MAPK, PI3K-AKT, and Rho/ROCK signaling, are involved in the regulation of MSC proliferation and osteogenic differentiation [49,50].

BMP ligands, such as BMP2, BMP4, BMP6, and BMP7, are crucial for osteogenesis and developmental processes [49]. Like TGF-β, BMP ligands undergo processing to form homodimers through disulfide bonds. Some BMP subunits can also form heterodimers, such as BMP2/7, BMP2/6, and BMP4/7, which exhibit enhanced biological activity in specific contexts [51]. BMP signaling is initiated when BMP ligands bind to type I receptors (e.g., ALK2, ALK3, ALK6) and type II receptors (e.g., BMPRII, ActRII, ActRIIB), activating both the canonical SMAD-dependent pathway [22,27,50] and non-canonical pathways, including TAK1-MAPK, PI3K-AKT, mTOR, and NF-κB [22,49,52]. These signaling pathways collectively regulate MSC osteogenesis and bone formation.

### 3.2. Effects of TGF-β/BMP Signaling on Osteogenesis

SMAD proteins are pivotal downstream effectors in the TGF-β/BMP signaling pathways, categorized into receptor-regulated R-SMADs, common mediator Co-SMAD, and inhibitory I-SMADs. In R-SMADs, SMAD2 and SMAD3 primarily mediate TGF-β signaling, while SMAD1, SMAD5, and SMAD8 are key mediators of BMP signaling. Upon receptor activation by TGF-β or BMP ligands, R-SMADs are phosphorylated, form complexes with Co-SMAD (SMAD4), and translocate to the nucleus to regulate the transcription of osteogenic target genes. I-SMADs, including SMAD6 and SMAD7, negatively regulate these pathways by inhibiting R-SMAD phosphorylation, competing for receptor binding, or facilitating R-SMAD degradation through E3 ubiquitin ligases such as Smurf1/2 [27,50,52].

**Figure 3:**
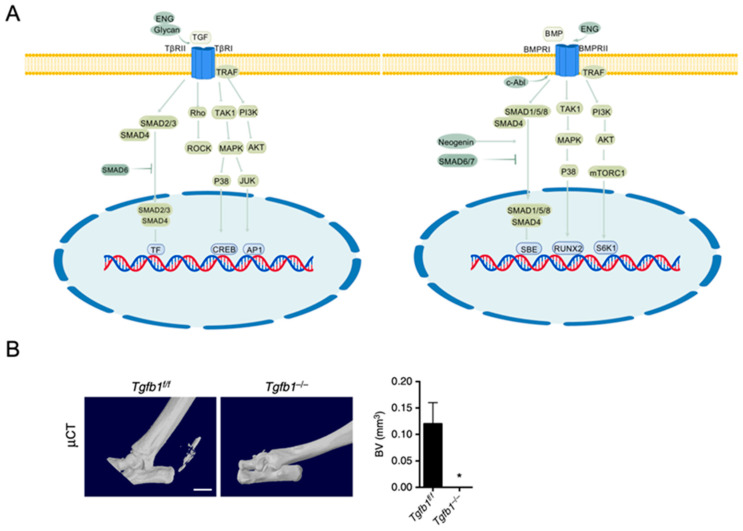
TGF-β/BMP Signaling in MSC osteogenesis. (**A**) Regulation of MSC osteogenic differentiation by TGF-β/BMP Signaling. TGF = transforming growth factor; ENG = endoglin; TRAF = TNF receptor-associated factor; TβRI = TGF-β receptor I; TβRII = TGF-β receptor II; PI3K = phosphoinositol-3 kinase; AKT = protein kinase B; TAK1 = transforming growth factor-β activated kinase 1; MAPK = mitogen-activated protein kinase; JUK = c-Jun N-terminal kinase; ROCK = rho-associated protein kinase; TF = transcription factor; CREB = cAMP response element-binding protein; AP1 = activator protein 1; BMP = bone morphogenetic protein; BMPRI = bone morphogenetic protein receptor I; BMPRII = bone morphogenetic protein receptor II; mTORC1 = mammalian target of rapamycin complex 1; c-Abl = abelson tyrosine-protein kinase 1; SBE = SMAD-binding element; RUNX2 = runt-related transcription factor 2; S6K1 = ribosomal protein S6 kinase 1. The figures are created with BioGDP.com. (**B**) HO formation was absent in the LysM-cre::Tgfb1flox/flox mouse model (Tgfb1^−/−^) after Achilles tendon puncture. * *p* < 0.05 as determined by unpaired Student’s *t*-test. Reproduced from Wang X et al. [56] under the terms of the Creative Commons Attribution International License (CC BY-NC-ND).

TGF-β signaling plays a complex role in osteoblast differentiation. In later stages of osteoblast differentiation, SMAD2/3 was reported to inhibit osteogenesis by competing with RUNX2 for binding to coactivators CBP/p300. TGF-β1 counteracts the effects of BMP2, suppressing its expression and that of other osteogenic genes (e.g., Col1a1, Alp, Ocn) [50]. On the other hand, TGF-β signaling has been shown to promote both proliferation and osteogenic differentiation in the early stages of MSC differentiation. For example, β-glucan enhances TGF-β binding to the TGFBR2-TGFBR1 complex, promoting osteogenesis [50]. Mice with impaired TGF-β signaling, including Tgfb1 knockout mice, MSC-specific Tgfbr2 knockout mice, Alk5 knockout mice, and Smad3 knockout mice, exhibit significant bone loss and a decrease in osteoblast numbers [49]. In humans, gain-of-function mutations in TGFB1 are associated with Camurati-Engelmann disease, characterized by sclerosis of long bones and cranial bones; mice carrying the same Tgfb1 mutation exhibit similar phenotypes observed in humans [49]. In the ECM, TGF-β is usually bound to latency-associated proteins (LAP) in an inactive complex. During tissue injury or remodeling, osteoclasts in the bone matrix cleave LAP to activate TGF-β. The active TGF-β then binds to TβRI and mediates signaling through SMAD, transiently recruiting perivascular MSCs to the recently resorbed bone surface for osteoblast differentiation and new bone formation [53,54,55]. TGF-β, released from the ECM by osteoclastic bone resorption after the onset of heterotopic ossification (HO), has been reported as crucial in recruiting MSCs for ectopic bone formation (Figure 3) [56]. Additionally, under specific conditions, TGF-β signaling promotes osteogenesis through non-canonical pathways, such as ERK and p38 signaling [57].

BMP signaling is a critical regulator of cartilage and bone formation during normal embryonic development. It promotes the expression of osteogenic genes by interacting with key transcription factors through SMAD1/5/8 [48,52]. BMP2 and BMP7 can induce osteoblast differentiation in MSCs from different origins. Bmp5 mutant short-eared mice exhibit multiple skeletal defects, including abnormalities in the sternum, ribs, and sixth cervical vertebra, as well as impaired fracture healing. Bmp7-deficient mice display skeletal changes in the ribs, hind limbs, and skull, while Bmp6 mutant mice show delayed sternum ossification [52]. In a Neogenin-deficient mouse model, endochondral ossification was impaired due to the lack of Smad1/5/8 signaling activation and subsequent RUNX2 expression [58]. Additionally, non-receptor tyrosine kinase c-Abl knockout mice exhibit perinatal lethality, growth retardation, and osteoporosis. This is due to the impaired BMP signaling through phosphorylation of BMPRIA, affecting both the canonical Smad1/5/8 and non-canonical Erk1/2 pathways to influence MSC proliferation and differentiation [53,59]. Inhibition of ubiquitin-mediated degradation of I-SMADs (e.g., SMAD6/7) further enhances BMP signaling activity [22], underscoring the pivotal role of canonical BMP signaling in osteogenic differentiation. ENG (Endoglin), a transmembrane glycoprotein primarily expressed in endothelial cells, is essential for angiogenesis and skeletal development. It binds TGF-β1/3 to facilitate TGF-β signaling through TβRII and also aids in BMP2 and BMP7 receptor binding, thereby enhancing BMP signal transduction. Additionally, evidence suggests that ENG upregulates BMP4 expression, indicating that TGF-β and BMP signaling pathways act synergistically to maintain bone homeostasis [50,60,61].

Non-canonical TGF-β/BMP signaling pathways promote osteogenesis independent of the SMAD pathway. Upon binding to their respective receptors, TGF-β or BMP activates TAK1 through TNF receptor-associated factor (TRAF)-mediated polyubiquitination, subsequently triggering the MAPK or PI3K signaling cascades. These pathways ultimately drive osteoblastic differentiation and maturation by regulating progenitor cell enrichment and early differentiation [22,27,49,52]. Extracellular superoxide dismutase (SOD3) modulates FLT1 (VEGFR1) via PI3K/AKT and MAPK pathways, suppressing MSC adipogenesis while promoting osteogenesis, thereby influencing bone remodeling and accrual [62]. In the non-classical pathway, the mTOR pathway also plays a role in promoting osteogenesis. For example, reduced mTORC1 signaling in Bmpr1a (ALK3)-deficient mice leads to suppressed osteoblast activity [63]. However, in some instances, non-canonical pathways antagonize canonical signaling. BMPRII couples with RANK to activate NF-κB signaling, and NF-κB interacts with SMAD4 to inhibit its transcriptional activity, thereby suppressing BMP2-induced bone formation [49]. Similarly, in cultured human MSCs, inhibition of the Rho/ROCK signaling pathway enhances Smad2/3 signaling under TGF-β3 stimulation [64,65]. These findings highlight the complex interplay between canonical and non-canonical TGF-β/BMP pathways in regulating osteogenesis (Figure 3).

### 3.3. Applications of TGF-β and BMP Signaling Pathways in Bone Regeneration

Given their pivotal roles in MSC osteogenic differentiation, TGF-β, and BMP signaling pathways are potential targets for bone regeneration. For instance, injectable TCP/chitosan (TC) composites combined with platelet-rich plasma (PRP) enhance MSC osteogenesis by upregulating TGF-β expression, demonstrating significant bone regeneration capacity when injected into goat tibial defects [64]. Due to hyperglycemia and oxidative stress, bone regeneration defects in diabetic patients remain a significant challenge. Scaffolds decorated with nanoscale ceria (nCe-scaffolds) enhanced cell adhesion and increased integrin expression, activating the TGF-β-SMAD2/3 and TGF-β-p38 pathways in MSCs, thereby facilitating bone regeneration in a streptozotocin (STZ)-induced diabetic bone defect mouse model [66]. However, the short half-life of TGF-β proteins necessitates delivery systems for sustained release in regenerative medicine applications. Moreover, the widespread expression of TGF-β receptors across tissues poses challenges, as it can lead to uncontrollable side effects, presenting significant clinical hurdles.

In contrast, the clinical application of recombinant BMPs (rhBMPs) has made substantial progress. The U.S. Food and Drug Administration (FDA) has approved two BMP-based clinical products: purified collagen matrices infused with BMP-2 (Medtronic) or OP-1^®^ BMP-7 (Stryker Biotech) for treating long bone fractures and improving intervertebral disc regeneration (Table 1) [22]. To address the challenges of degradation and rapid clearance that lead to a short half-life, BMP proteins in in vivo studies are often combined with biocompatible carriers, such as collagen sponges, in bone tissue engineering [27,28,67,68].

## 4. PTH/PTHR Signaling Pathway

### 4.1. Overview of PTH/PTHR Signaling

PTH, secreted by the parathyroid glands, regulates calcium and phosphate metabolism through binding to the PTH1 receptor (PTH1R). Chronic high levels of PTH lead to bone resorption, but intermittent or pulse-like administration of PTH can promote bone formation by stimulating osteoblast activity [29]. The binding of PTH to its receptor follows the two-site binding model, where the amino (N)-terminal of the ligand binds to the receptor’s transmembrane core, triggering signal transduction, while the carboxyl (C)-terminal stabilizes the ligand-receptor complex by binding to the extracellular domain [69].

PTH exists in several variants, including splice isoforms and N- and C-terminal variants. The active fragment PTH (1–34) is commonly used in clinical treatments. A key variant is PTHrP (Parathyroid hormone-related protein), initially found in patients with hypercalcemia of malignancy and secreted by various cells in bone, cartilage, mammary glands, placenta, and skin. PTHrP shares structural similarities with PTH and binds to the same receptor, PTH1R, to exert similar effects [29,70]. As a paracrine/autocrine factor produced by osteoblasts and osteocytes, PTHrP can independently stimulate osteoblast differentiation and trabecular bone formation. However, excessive PTHrP secretion in cancers like lung, breast, and kidney can cause hypercalcemia [70].

PTH and PTHrP primarily act through parathyroid hormone receptor 1 (PTH1R) and parathyroid hormone receptor 2 (PTH2R). PTH1R, widely expressed in bone and kidneys, is a GPCR that activates cAMP/PKA and PLCβ/IP3/DAG pathways to regulate calcium levels and effector molecule expression. It also influences cell proliferation and differentiation via ERK1/2 MAPK [71,72]. PTH2R, mainly binding PTH, is expressed in the nervous and vascular systems, mediating cAMP signaling, though its functions are less defined [73,74]. By integrating these signaling pathways, PTH and PTHrP regulate various systemic functions.

### 4.2. Impact of PTH/PTHrP Signaling on Bone Homeostasis

**Figure 4:**
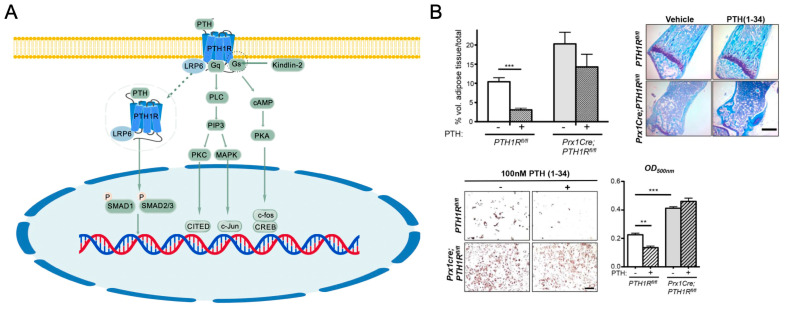
PTH/PTHR signaling pathway involved in MSC osteogenesis regulation. (**A**) PTH/PTHR signaling in MSC osteogenesis. PTH = parathyroid hormone; PTH1R = parathyroid hormone receptor 1; PLC = phospholipase C; cAMP = cyclic adenosine monophosphate; PIP3 = phosphatidylinositol-(3,4,5)-trisphosphate; PKA = protein kinase A; PKC = protein kinase C; MAPK = mitogen-activated protein kinase; LRP = low-density lipoprotein receptor-related protein; CITED = CBP/p300-interacting transactivator with ED-rich tail; CREB = cAMP response element-binding protein. The figures are created with BioGDP.com. (**B**) Control of bone marrow adipose tissue (BMAT) by PTH. Genetic ablation of the PTH/PTHrP receptor (PTH1R) in Prx1Cre-expressing MSCs caused diminished bone formation and increased BMAT. Daily administration of PTH (1–34) in control mice reduced BMAT and inhibited adipogenic differentiation. Scale bar: 500 μm. ** *p* < 0.01, *** *p* < 0.001 versus PTH1R^fl/fl^ under vehicle treatment. Reproduced with permission from Fan Y et al. [75].

PTH1R is expressed throughout all stages of osteoblast lineage differentiation, regulates osteoblast differentiation, proliferation, and survival, thereby promoting bone formation, increasing mass, and reducing fracture risk. Genetic deletion of the PTH/PTHrP receptor (PTH1R) in Prx1Cre-labeled MSCs resulted in low bone formation, increased bone resorption, and elevated bone marrow adipose tissue (BMAT). In contrast, daily injections of PTH (1–34) reduced BMAT levels in control mice, inhibited adipogenic differentiation, and directed the fate of BMSCs toward osteogenesis (Figure 4) [75]. Transgenic mice expressing a human mutant PTHR (HKrk-H223R) under a 2.3-kb α1(I) collagen promoter exhibited enhanced MSC differentiation into osteoblasts and increased trabecular bone volume, highlighting PTH1Rs anabolic role [76]. The H223R mutation in PTH1R promotes cAMP-independent signaling upon PTH or PTHrP stimulation, leading to Jansen metaphyseal chondrodysplasia (JMC), a rare disease characterized by preserved trabecular bone and excessive cortical bone loss [76]. In contrast, homozygous deletion of PTH1R in mice reduced trabecular bone volume and increased cortical bone thickness during fetal development, resembling Blomstrand chondrodysplasia (BOCD), a condition linked to complete PTH1R loss-of-function mutations [76,77].

Upon binding to PTH1R, PTH activates Gαs, triggering the cAMP/PKA pathway and phosphorylating CREB (cAMP response element-binding protein) and other transcription factors. Phosphorylated CREB then interacts with c-jun and c-fos to promote the expression of key osteogenesis-related genes. PKC can also be activated in this pathway and exert similar effects as PKA. Kindlin-2, the adhesion protein that assists in PTH and receptor binding, upregulates CREB phosphorylation and promotes MSC differentiation into osteoblasts, followed by osteoblast proliferation and differentiation. Kindlin-2 deletion completely abolished PTH effects on stimulation on bone volume, bone mineral density, and cortical thickness in ovariectomized mice [78]. Zinc finger proteins (ZFPs), the largest class of transcription factors in eukaryotic genomes, have been identified as critical regulators of osteogenesis and adipogenesis. PTH inhibits the expression of the Zfp467 via the PKA pathway, leading to nuclear translocation of NFκB1, which binds to the P2 promoter of Pth1r, promoting its transcription. PTH1R and ZFP467 form a feedback loop to enhance PTH-induced osteogenesis [79].

Intermittent administration of PTH (1–34) in MSCs increases PKCδ expression, which induces Runx2 expression, upregulates ALP and COL1a1 expression, and promotes osteoblastic differentiation [30,31]. Continuous PTH stimulation promotes aerobic glycolysis, which activates PI3K/mTORC2 signaling while inhibiting mitochondrial glucose metabolism (TCA), thereby reducing trabecular bone formation [80]. On the other hand, PTH also increases the expression of IGF and dual-specificity phosphatases, triggering IGFIR and EGFR activation, which subsequently activate AKT, P38, and c-Jun N-terminal kinase (JNK-MAPK) signaling pathways [80]. Furthermore, downstream targets of PTH/PTH1R signaling, including IGF-1, FGF-2, RANKL, and sclerostin, also play crucial roles in promoting MSC differentiation into osteoblasts [75].

The ternary complex formed by PTH, PTH1R, and LRP6 can be internalized into cells, enhancing the binding affinity of BMP to its receptor, thereby promoting BMP-Smad signaling and MSC differentiation. This complex also facilitates the rapid phosphorylation of LRP6, recruits axin to LRP6, and stabilizes β-catenin, which activates the Wnt signaling pathway to promote osteoblastic differentiation [54,80,81]. PTH regulates TGF-β signaling by forming a complex with the TGF-β type II receptor (TbetaRII), where TbetaRII phosphorylates the cytoplasmic domain of PTH1R, regulating the endocytosis of the complex. Conditional knockout of TbetaRII in mouse osteoblasts results in high bone mass, increased trabecular bone, and decreased cortical bone, resembling the bone phenotype of mice with high expression of active PTH1R. Moreover, knocking out PTH1R to disrupt PTH signaling rescued the bone phenotype of TbetaRII knockout mice, indicating that PTH and TGF-β signaling pathways act antagonistically [82].

PTH/PTHrP signaling pathway maintains calcium-phosphate balance, bone health, and endocrine homeostasis through the aforementioned pathways. These functions are widely used in developing anabolic therapies for osteoporosis. PTH (1–34) (teriparatide) has been shown effective in treating postmenopausal osteoporosis. PTHrP analogs, like abaloparatide, which modifies five residues of PTHrP (1–34) from positions 22 to 34, show a more favorable effect on bone formation with a smaller increase in bone turnover markers compared to teriparatide in osteoporosis clinical studies (Table 1) [29]. The PTH/PTHrP signaling pathway is essential for bone metabolism and holds promise for osteoporosis treatment.

### 4.3. Application of PTH/PTHrP Signaling Pathway in MSC-Based Bone Tissue Regeneration

In bone injuries, PTH promotes MSC migration to damaged areas, which is essential for regeneration [83]. In rat and pig models with vertebral defects, systemic injection of exogenous MSCs combined with intermittent PTH administration resulted in more effective repair. Labeled MSCs were primarily found in the bone defect area and in newly formed bone tissue, supporting the hypothesis that PTH enhances MSC migration to defective sites [83]. PTH1–34 (teriparatide) and PTHrP promote MSC osteogenic differentiation while inhibiting apoptosis in mature osteoblasts and osteocytes [1,84]. Additionally, PTHrP suppresses PPARγ activity during MSC differentiation, thereby inhibiting adipogenic differentiation and promoting osteogenic differentiation [75].

PTH1–34 treatment has been shown to promote bone formation in long bone fractures, cortical bone defects (membranous ossification), severe bone defects, and spinal joint fixation models [85]. In bone tissue engineering, local PTH1–34 therapy with scaffold materials offers advantages over intermittent systemic treatment by achieving similar effects without the need for repeated injections. For example, scaffolds with covalently bound PTH1–34 peptides, modified with arginine-glycine-aspartate (RGD) sequences, enhanced bone regeneration in canine hip bone defects [86]. Similarly, scaffolds made from transglutaminase substrate-bound fibrin and PTH1–34 fragments promoted bone regeneration in cylindrical defects (8 mm diameter, 13 mm depth) in the femur and humerus of ewes [87]. However, the optimal dosing regimen for PTH1–34 in bone repair remains a critical issue. While PTH1–34 treatment appears ideal for bone healing, considerations regarding potential risks, such as osteosarcoma, along with the high cost of the medication and the inconvenience of daily subcutaneous injections, should be taken into account (Figure 4).

## 5. Hedgehog Signaling Pathway

### 5.1. Overview of Hedgehog Signaling

The Hedgehog (Hh) signaling pathway is a critical regulator of cellular differentiation and includes three primary ligands: Sonic Hedgehog (SHH), Indian Hedgehog (IHH), and Desert Hedgehog (DHH). Among these, SHH and IHH share overlapping functions in various tissues. SHH primarily regulates osteoblast differentiation and function, playing a pivotal role in bone development. In adults, SHH signaling remains crucial for bone repair and regeneration. IHH, on the other hand, is primarily involved in endochondral ossification, modulating bone growth and morphology. DHH is mainly restricted to the gonads, regulating granulosa cell and Sertoli cell functions [88].

The Hh signaling pathway operates through classical and non-classical mechanisms. In the classical pathway, insoluble Hh ligands are activated via modifications with cholesterol (C-terminal) and palmitate (N-terminal). These modified ligands are released from secreting cells via the transmembrane protein Dispatched (DISP), often associated with lipoproteins or extracellular vesicles. Released Hh ligands bind to the Patched (PTCH) receptor on target cells, relieving the inhibition of Smoothened (SMO), thereby activating downstream signaling. This leads to the activation of the Gli transcription factor family (Gli1, Gli2, Gli3), which translocate to the nucleus to regulate the expression of osteogenesis-related target genes [34,88,89]. Non-classical Hh signaling, in contrast, operates through Gli-independent mechanisms. This pathway can regulate cellular chemotaxis and migration via actin cytoskeleton rearrangement and promote cell proliferation through calcium-dependent activation of ERK [90].

### 5.2. Hh Signaling Regulates Bone Tissue Development and Homeostasis

SHH is a critical regulator of cellular differentiation, particularly exhibiting pro-osteogenic and anti-adipogenic properties in MSCs [34]. miR-196a stimulation in osteoporotic mouse BMSCs upregulates Smo expression, along with increased ALP level and activity and enhanced expression of RUN2, OPN, OCN, and tartrate-resistant acid phosphatase [91]. Overexpression of Shh results in mandibular enlargement [92], while mutations in Ift88, a protein essential for cilia function, cause downregulation of Hh signaling and slight mandibular shortening. Similarly, Smo mutations lead to pronounced mandibular shortening [93]. Transgenic mice lacking Shh exhibit severe skeletal development abnormalities, growth retardation, and the absence of vertebrae and distal limb structures, emphasizing its crucial role in bone development and osteogenesis [94,95].

IHH is a critical factor in embryonic and neonatal skeletal development. In addition to regulating chondrocyte and osteoblast proliferation and differentiation, IHH also plays a key role in vascularization within the newly formed skeleton. Ihh-deficient animals exhibit shortened limbs and a lack of mineralized bone structures [95,96]. Two missense mutations (P46L, V190A) in the amino-terminal signaling domain of the IHH gene cause scapular dysplasia, characterized by short stature and shortened limbs, inherited in an autosomal recessive manner [97]. IHH stimulation promotes perichondral and columnar chondrocyte differentiation, regulating cartilage development and preventing premature hypertrophic differentiation, while IHH overexpression accelerates column elongation and perichondral chondrocyte differentiation [98].

Hh proteins themselves can serve as targets. Hedgehog acyltransferase (HHAT) promotes the Hh palmitoylation, thereby enhancing Hh signaling. In HHAT-deficient mice, Hh secretion is reduced. These embryos exhibit widespread apoptosis in the forebrain and craniofacial mesenchyme, underdeveloped frontal-nasal, mid-lateral, maxillary, and mandibular bones, and reduced chondrogenesis and osteogenesis [93,99]. Smo is a key target in the Hh signaling pathway. Purmorphamine, a purine derivative, activates Smo, thereby lowering cAMP levels and promoting Gli2 and Gli3 translocation to the nucleus, enhancing Hh target gene expression and mesenchymal progenitor proliferation [100,101,102]. Cholesterylamine, a cholesterol derivative, also binds to Smo and activates Hh signaling, inhibiting PPARγ, thereby blocking MSC adipogenesis and promoting osteogenesis [101,103]. A novel Hh co-receptor, SLT and neurotrophic tyrosine receptor kinase-like protein-5 (slitrk5), has been identified to bind SHH and PTCH1, inhibiting Hh signaling. Due to its specificity and selective expression in osteoblasts, slitrk5 may serve as a new target for promoting osteoblast differentiation via the Hh pathway [104]. Among the Gli transcription factors, only Gli1 acts as a full-length activator, while Gli2 and Gli3 regulate the pathway positively or negatively, depending on modifications. When transcription is inactive, Gli3 usually functions as a repressor [90]. In vitro, Gli3 promotes osteoblast differentiation in C3H10T1/2 cells by increasing ALP activity and expression of RUNX2, OPN, and OCN while inhibiting adipocyte differentiation. Additionally, Shh/Gli3-induced osteoblast differentiation is synergistic with BMP2 and enhances FGF18 expression, forming a positive feedback loop that promotes osteogenesis [105]. Hh signaling activity decreases with aging. Inactivation of Smo results in reduced bone formation and increased bone marrow adiposity, key features of osteoporosis [106], while oxidative stress inhibits the expression of Gli1 and Patched1, impairing osteogenesis [107], which may contribute to age-related osteoporosis.

### 5.3. Hh Signaling in MSC-Mediated Bone Tissue Engineering

The role of the Hh signaling pathway in osteogenic induction has been confirmed in in vitro studies across various cell types and experimental conditions. IHH overexpression in BMSCs and C3H10T1/2 cells significantly increases osteogenic marker expression, ALP activity, and mineralization. SHH stimulates osteogenic differentiation through both RUNX2-dependent and independent mechanisms in early osteoblasts [108].

Abnormal bone repair in diabetic (Db) mice is reported due to the high serum levels of inflammatory cytokines, which directly inhibit IHH expression in MSCs, impairing healing. The precise delivery of purified IHH to fracture sites using biodegradable, sustained-release polyethylene glycol (PEG) hydrogels reversed this defect and rescued bone healing in Db mice [109]. Purmorphamine activates the Hh signaling pathway by stimulating Smo, leading to upregulation of the downstream target gene GLI1 and the Wnt/β-catenin pathway, promoting BMSC osteogenic differentiation and bone formation, both in 2D and 3D modules created using the Regenova^®^ system (Cyfuse Biomedical KK, Tokyo, Japan) [110].

**Figure 5:**
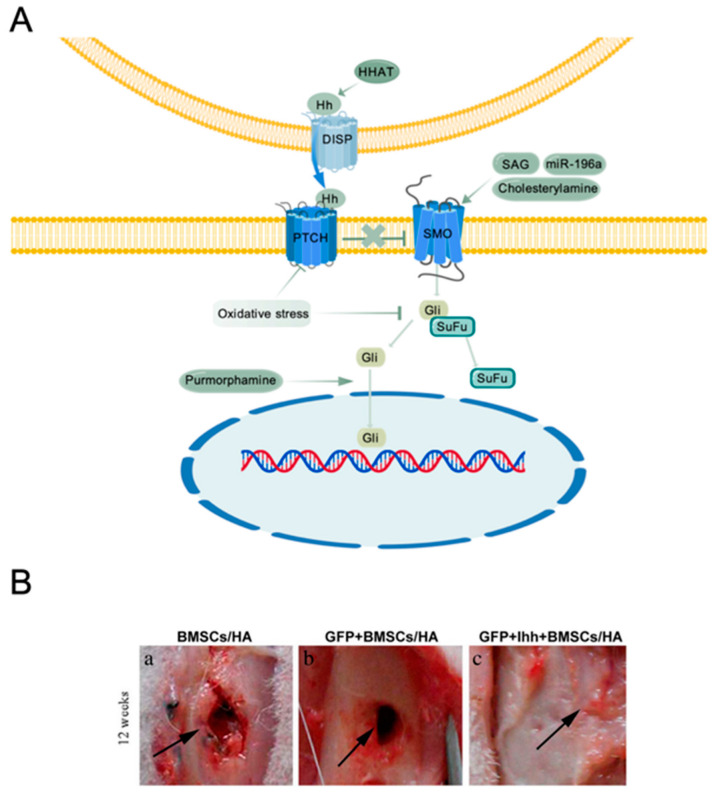
Role of Hedgehog (Hh) signaling in MSC osteogenic differentiation. (**A**) Hh Signaling in MSC osteogenesis. HHAT = hedgehog acyltransferase; DISP = dispatched; PTCH = patched; SMO = smoothened; SAG = smoothened agonist; Gli = glioma-associated oncogene; SuFu = suppressor of fused; miR-19 = microRNA-19. The figures are created with BioGDP.com. (**B**) The implanted Ihh/MSCs/hydroxyapatite (HA) scaffold complex enhanced bone repair in a rabbit tibial defect model. Arrows in all panels points to the injury sites. Reproduced from Zou S et al. [112] under the terms of the Creative Commons Attribution International License (CC BY-NC-ND).

Transplantation of MSCs overexpressing the N-terminal fragment of SHH (ShhN) significantly improved cell survival and enhanced bone regeneration at the femoral defect site in mice [111]. In a critical-sized rabbit cranial defect model, the implantation of SHH-N overexpressing MSCs also promoted bone formation [82]. The IHH/MSC/scaffold composite implanted into the rabbit tibial defect site promoted bone repair [88,112]. Cholesterylamine enhances Hh signaling by binding to Smo, which in turn affects the function of Hh ligands (e.g., SHH) and receptors (e.g., PTCH1). Implanting cholesterylamine-loaded scaffolds into a rat plantar bone defect model significantly improved fracture healing and new bone formation [113]. Moreover, biomaterials used in bone tissue engineering, e.g., bioactive glass-ceramics (BGC), TiO_2_ nanotube micro/nano-textured topography (MNT), fluorophosphate-modified PCL nanofibers, and nanoscale hydroxyapatite-coated titanium (nHA), have been shown to stimulate MSC differentiation into osteoblasts and enhance bone formation through Hh signaling [101]. However, due to its involvement in tumorigenesis, treatment strategies must balance safety and efficacy for successful clinical use (Figure 5).

## 6. IGF Signaling Pathway

### 6.1. Overview of IGF Signaling

IGF-1, regulated by growth hormone (GH), is mainly secreted by the liver into the bloodstream and locally by osteoblasts and chondrocytes, regulating bone formation and remodeling via autocrine and paracrine mechanisms. IGF-2, expressed in tissues like the liver, placenta, and bone during embryonic development, supports early stages of bone formation through systemic or local secretion [114,115]. IGF-1 and IGF-2 initiate signaling by binding to their respective receptors. Both IGF-1 and IGF-2 can bind to IGF-1R, a receptor tyrosine kinase (RTK) composed of two α subunits and two β subunits linked by disulfide bonds. The extracellular α subunits form a binding pocket, while the β subunits contain an intracellular tyrosine kinase domain [116,117]. Ligand binding triggers IGF-1R autophosphorylation, activating downstream PI3K/AKT and MAPK/ERK pathways [118], promoting osteoblast proliferation, survival, differentiation, and matrix synthesis. In addition, IGF-2 can bind to insulin receptor isoform A (IR-A), playing a unique role in embryonic and early bone formation. Unlike IGF-1R, IGF-2R functions as a multifunctional scavenger receptor, primarily regulating IGF-2 activity by internalizing and degrading it. This process limits IGF-2s interaction with IGF-1R, indirectly suppressing downstream signaling [119].

IGFBPs regulate IGF signaling by forming complexes with IGFs and the acid-labile subunit (ALS). These complexes modulate IGF bioavailability and activity by restricting IGFs interaction with receptors (e.g., IGF-1R), stabilizing IGFs in circulation, and prolonging their half-life. Additionally, IGFBPs can release IGFs in specific locations, enabling localized signaling and fine-tuning the duration and intensity of IGF-mediated effects [117,120].

### 6.2. IGF Pathway in Bone Development and Homeostasis

Upon binding to IGF-1R, IGF-1 induces autophosphorylation of the receptor, which recruits and phosphorylates insulin receptor substrate-1 (IRS-1). This activation leads to the PI3K pathway, where PI3K converts PIP2 into PIP3, triggering the activation of PDK1 and AKT. Activated AKT inhibits MSC apoptosis. Additionally, IGF-1 binding phosphorylates Src homology collagen (SHC), which activates the Ras-Raf-MEK-ERK pathway. Activated ERK translocates to the nucleus to regulate the expression of genes involved in MSC proliferation and osteogenic differentiation [116]. IGF-1 also promotes protein synthesis and pre-osteoblast differentiation through the PI3K/AKT/mTOR pathway [117]. In vivo, IGF-1 deficiency results in severe growth retardation and significantly reduced bone density. Adult IGF-1^−/−^ mice exhibit impaired bone formation during remodeling, with a marked reduction in mature osteoblasts on bone remodeling surfaces. Conversely, mice overexpressing IGF-1 in mature osteoblasts show enhanced bone formation, with increased trabecular and cortical bone volume [117]. Serum response factor (SRF) enhances IGF-PI3K/AKT signaling in osteoblasts, increasing Runx2 DNA-binding activity and promoting osteogenesis. Mice with osteoblast-specific SRF deficiency showed a reduction in bone mass starting at 9 weeks of age [118]. Knockout of IGF-1R results in severe growth retardation and bone defects, confirming its essential role in embryonic development [121]. In Kashin-Beck disease, IGF-1R inhibition leads to chondrocyte death and matrix degradation, emphasizing its importance in chondrocyte regulation [122]. AMPKα1, a key regulator of cellular energy metabolism, plays a critical role in the osteogenesis of aged MSCs through the AMPKα1/IGF-1/CREB axis. AMPKα1 stimulates phosphorylation of IGF-1R and downstream signaling, promoting IGF-1 transcription in a positive feedback loop [123]. Additionally, insulin receptor substrates IRS-1 and IRS-2 are activated by IGF-1R signaling. In mice, IRS-1 deficiency in osteoblasts reduces bone formation and resorption, while IRS-2 knockout leads to decreased bone formation and increased resorption [124,125].

IGF-2 is essential for embryonic development and early bone formation, with its expression regulated by imprinted genes. During fetal growth, IGF-2 levels are higher than IGF-1 and promote osteoprogenitor cell proliferation and differentiation. In adults, while serum IGF-2 levels are elevated, its function is reduced, playing a secondary role in bone repair [114]. Its unique function may be linked to its preferential binding to receptors like IR-A, influencing different developmental stages and physiological conditions [114].

The IGFBP family is a highly conserved protein family, consisting of seven members. IGFBP-2, by binding to the cell surface receptor RPTPβ, inactivates the AKT inhibitor PTEN, thereby activating the AKT pathway. This highlights the ability of IGF-I to stimulate the AKT pathway. The IGFBP-2/RPTPβ signaling pathway works in coordination with the IGF-I receptor signaling pathway to promote osteoblast differentiation and bone formation [126]. IGFBP-2 knockout mice exhibit reduced bone mass and trabecular bone volume/total volume ratios [126]. IGFBP-3 promotes the deposition of IGF-1 in the bone matrix, facilitating its role in bone remodeling [127]. IGFBP-4 exerts an inhibitory effect by reducing the bioavailability of IGF-1. Pregnancy-associated plasma protein-A (PAPP-A), a metalloproteinase, activates the IGF signaling pathway by cleaving IGFBP-4, thereby regulating osteoblastic differentiation and bone formation in MSCs [128]. Mice lacking IGFBP-4 show a 10–15% reduction in body size before and after birth compared to wild-type mice, while osteoblast-specific overexpression of IGFBP-4 reduces bone turnover and inhibits bone growth [129,130]. IGFBP-5 partially promotes bone formation through an IGF-1-dependent mechanism [131]. Long-term treatment with rhIGF-I/IGFBP-5 complexes for 8 weeks in ovariectomized mice or mature rats significantly increased cortical thickness, area, and density of femoral shafts or tibial ossification [132].

### 6.3. IGF Pathway in Bone Tissue Engineering Applications

**Figure 6:**
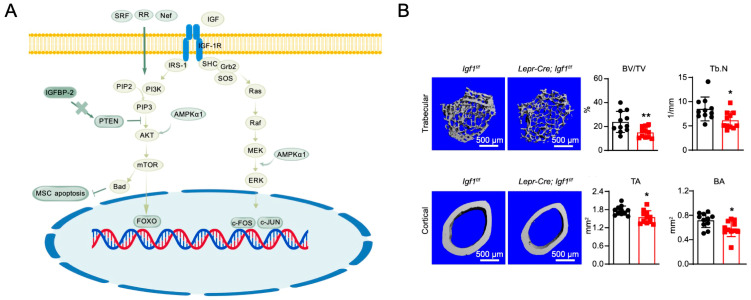
(**A**) IGF pathway regulating MSC osteogenic differentiation. IGF = insulin-like growth factor; IGF-1R = insulin-like growth factor 1 receptor; IRS = insulin receptor substrate-1; SHC = src homology collagen; Grb2 = growth factor receptor-bound protein 2; SOS = son of sevenless; Raf = rapidly accelerated fibrosarcoma; MEK = mitogen-activated protein kinase kinase; ERK = extracellular signal-regulated kinase; PI3K = phosphoinositol-3 kinase; PIP3 = phosphatidylinositol-(3,4,5)-trisphosphate; PIP2 = phosphatidylinositol-(4,5)-bisphosphate; AKT = protein kinase B; mTOR = mammalian target of rapamycin; SRF = serum response factor; RR = rehmanniae radix praeparata; AMPKα1 = amp-activated protein kinase α1; IGFBP-2 = insulin-like growth factor binding protein 2; FOXO = forkhead box O. The figures are created with BioGDP.com. (**B**) Deletion of Igf1 from BMSCs impairs bone maintenance by reducing bone formation. Data represent mean ± SD (* *p* < 0.05, ** *p* < 0.01). Reproduced from Wang J et al. [135] under the terms of the Creative Commons Attribution International License (CC BY-NC-ND).

The IGF pathway plays a key role in bone tissue engineering and repair. However, the short half-life of recombinant IGF-1 (rhIGF-1) limits its long-term effectiveness. To address this, rhIGF-1-loaded double-layer microspheres, prepared using poly (lactic acid) and hydroxyapatite as materials and the oil-in-water-oil (*w*/*o*/*w*) technique, have shown good cell compatibility and long-term delivery efficacy. When implanted subcutaneously in C57BL/6 mice, these microspheres effectively delivered rhIGF-1, improving the bone mass and the number of OCN-positive osteoblasts on the bone surface, offering a strategy for preventing bone loss and delaying osteoporosis [133,134]. BMSCs and platelet-rich plasma (PRP), enriched with megakaryocytes/platelets (MKs/PLTs), promote bone repair by upregulating IGF-1 expression. It significantly enhances ALP activity and osteogenic differentiation in BMSCs and stimulates bone formation when transplanted into critical-sized palatal defects in mice (Figure 6) [135]. In bone tissue engineering, hydroxyapatite-chitosan-gelatin scaffolds provide an ideal platform for regenerative medicine. Chitosan scaffolds enhance MSC proliferation and osteogenic activity by supporting sustained release of IGFs and providing mechanical strength, modulating platelet activity at the wound site, making them a promising material for regenerative transplantation [136].

In the mouse femoral defect model, ECM secreted by hUCMSCs (hUCMSC-ECM) improved the proliferation of endogenous MSCs and enhanced bone regeneration. IGFBP-3, the most abundantly deposited cytokine in hUCMSC-ECM, promoted hBMSC migration through TβRI/II and CCR2-dependent mechanisms, thereby enhancing its therapeutic potential in bone regeneration [137]. The PI3K/Akt signaling pathway downstream of IGF was found to be important in the osteogenesis of BMSCs in a hydroxyapatite/β-TCP scaffold, which effectively promoted bone repair and regeneration in a cranial critical-sized bone defect model [138]. Neferine (Nef), a natural bisbenzylisoquinoline alkaloid extracted from lotus seeds, was used to construct a BGP/PLGA/Nef composite material. By activating the IGF-1/PI3K/Akt/mTOR signaling pathway, it effectively promoted new bone formation and maturation in a rat calvarial defect model, achieving complete bone bridging and coverage of the defect area [139]. Plant-derived compounds such as catalpol (CAT), acteoside (ACT), and echinacoside (ECH) extracted from Rehmanniae Radix praeparata (RR) also enhance IGF-1 production in osteoblasts, regulate the IGF-1/PI3K/mTOR signaling pathway, and upregulate osteogenic gene expression to promote bone formation [140]. These studies collectively highlight the potential applications of IGF pathway-targeted strategies in bone tissue engineering (Figure 6). Therefore, precautions should be taken when stimulating IGF production for bone repair to ensure safety [141].

## 7. Innovative Approaches Targeting MSC Signaling Pathways

Recent advances in MSC-based osteogenic therapies have highlighted emerging trends leveraging MSC-associated signaling pathways. Bioinformatics-driven pathway analysis and drug discovery have opened new avenues for identifying key regulatory factors and therapeutic targets involved in MSC osteogenesis. These computational approaches enable the integration of large datasets, offering deeper insights into pathway interactions and their roles in bone diseases and regeneration. For instance, bioinformatic analysis of gene expression changes at different time points during BM-MSC osteogenesis has revealed the interplay between the stem cell niche, ECM regulation, and key signaling pathways such as Wnt, TGF/BMP, and IGF, along with their downstream factors like PI3K/AKT and NF-κB, underscoring their critical roles [142]. By screening differentially expressed genes and miRNAs involved in MSC differentiation into osteoblasts versus adipocytes, enriched pathways (e.g., glutathione metabolism) and pivotal regulatory factors (e.g., miR-203, NEGR1) have been identified, providing a framework for constructing potential regulatory networks. This lays the foundation for exploring the specific mechanisms of key signaling pathways and identifying potential targets for therapeutic and regenerative applications [143]. Bioinformatics tools can also elucidate transcriptional regulation during osteoblast differentiation. Techniques such as ATAC-seq can be employed to investigate how enhancers regulate gene expression, offering valuable insights into the transcriptional landscape of osteogenesis [144].

In addition, nanotechnology-based drug delivery systems have garnered attention for their ability to specifically target signaling pathways [145]. Current limitations in bone disease treatments include low targeting efficiency, adverse effects on other organs and tissues, short plasma half-life, and poor physicochemical stability in biodistribution. Targeted delivery using nanomaterials offers a potential solution to enhance therapeutic efficacy and reduce side effects. For example, chitosan (CS)-based nanoparticles are effective gene carriers. The combination of CS nanoparticles with polyethyleneimine (PEI) has been shown to improve the delivery of the human BMP2 gene (hBMP2), promoting greater bone formation in rat bone defect models. Additionally, CS modified with sulfate groups can enhance the bioactivity of BMP-2 in bone repair by improving its encapsulation and sustained release [146,147]. Peptide amphiphiles (PA) can facilitate the controlled delivery of growth factors within synthetic grafts by providing ligand-specific binding platforms. Heparin-binding PA binds to the heparan sulfate domain of BMP-2, reducing the proportion of BMP-2 released within 24 h (22.7%) and significantly increasing the volume of new bone formation in rat defect gaps when applied with collagen sponges [148]. Approaches utilizing peptide ligands, aptamer ligands, and small molecule ligands for cellular delivery, as well as subcellular-targeted nanotechnologies, such as targeting autophagosomes, endoplasmic reticulum (ER), or lysosomes, improve the precision and efficiency of delivering bioactive molecules—such as growth factors; small molecules; and nucleic acids—directly to MSCs; thereby enhancing therapeutic outcomes [149,150]. Incorporating these innovative strategies into MSC-related research can fine-tune key signaling pathways, maximizing therapeutic potential in bone disease and bone tissue engineering.

## 8. Conclusions and Future Directions

**Figure 7:**
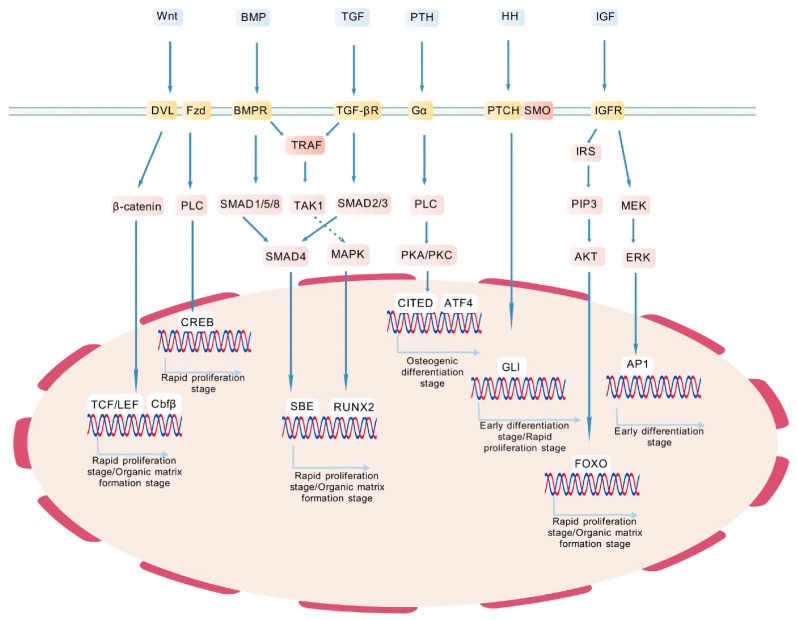
Overview of the pathway regulating MSC osteogenic differentiation. Wnt = wingless/int; BMP = bone morphogenetic protein; TGF = transforming growth factor; PTH = parathyroid hormone; HH = hedgehog; IGF = insulin-like growth factor; DVL = dishevelled; FZD = frizzled; BMPR = BMP receptor; TRAF = TNF receptor-associated factor; TGF-βR = TGF-β receptor; Gα = G protein alpha subunit; PTCH = patched; SMO = smoothened; IGFR = IGF receptor; PLC = phospholipase C; TAK1 = TGF-β-activated kinase 1; IRS = insulin receptor substrate; PIP3 = phosphatidylinositol (3,4,5)-trisphosphate; MEK = MAPK/ERK kinase; MAPK = mitogen-activated protein kinase; PKA = protein kinase A; PKC = protein kinase C; AKT = protein kinase B; ERK = extracellular signal-regulated kinase; CREB = cAMP response element-binding protein; CITED = cbp/p300 interacting transactivator with ED-rich tail; ATF4 = activating transcription factor 4; TCF/LEF = T cell factor/lymphoid enhancer factor; Cbfbeta = core binding factor β; SBE = SMAD binding element; RUNX2 = runt-related transcription factor 2; GLI = GLI family zinc finger; AP1 = activator protein 1; FOXO = forkhead box O. The figures are created with BioGDP.com.

In conclusion, the Wnt, TGF-β/BMP, PTH, Hh, and IGF signaling pathways are essential for skeletal development and homeostasis (Figure 7). However, their intricate interactions and dynamic regulation under various physiological and pathological conditions remain incompletely understood, posing challenges for their therapeutic application. Future research could further explore the temporal and spatial dynamics of these pathways in the skeletal system, which may help in the development of safer and more effective strategies for bone disease treatment.

As seed cells for bone tissue engineering, addressing MSC heterogeneity and enhancing their osteogenic potential are critical for effective application. Strategies such as isolating MSCs based on specific surface markers and optimized culture conditions can improve MSC uniformity and functionality. On the other hand, innovative biomaterials developed based on insights into critical signaling pathways provide powerful tools to promote MSC osteogenic differentiation. For example, optimizing scaffold microarchitecture can mimic the physiological bone environment, while the incorporation of nanomaterials, bioactive factors, ECM, and exosomes can interact with MSCs to enhance their responsiveness by modulating osteogenic signaling. Additionally, advanced genetic and epigenetic tools enable precise modulation of key pathways, further enhancing MSC differentiation. Emerging technologies, such as 3D culture systems and organoid models, offer accurate platforms for further investigating signaling influence on MSC behavior [151,152]. Finally, clinical studies assessing the long-term safety and efficacy of MSC-based therapies will be key to translating these basic research findings into effective treatments for bone defects. Addressing these challenges will pave the way for more precise, personalized strategies in bone regeneration therapies.

## Figures and Tables

**Table 1:** Signaling Pathways and Therapeutic Agents.

Signaling Pathway	Drug	Brand Name	Target Disease	Therapeutic Methods	Mechanisms of Action	Limitations and Risks
Wnt	Monoclonal antibody of SOST (Romosozumab)	Evenity (Manufacturer: Amgen Inc., Thousand Oaks, CA, USA; UCB Pharma, Brussels, Belgium)	Osteoporosis	Subcutaneous injection	Blocking sclerostin increases osteoblast activity and simultaneously decreases osteoclast activity.	Increased risk of cardiovascular events and bone-related side effects [24,26]
Wnt	Monoclonal antibody of SOST (Romosozumab)	APC+^®^ (Manufacturer: Amgen Inc., Thousand Oaks, CA, USA)	Osteoporosis	Local injection or implantation	Blocking sclerostin increases osteoblast activity and simultaneously decreases osteoclast activity.	Injection site reactions, risk of osteonecrosis of the jaw, and possible cardiovascular concerns [24,26]
Wnt	Fully human antibody of SOST (Setrusumab)	BPS804 (Manufacturer: Novartis, Basel, Switzerland)	OI	Subcutaneous injection	Blocking sclerostin improves bone density and reduces fracture risk in patients with OI.	Incomplete efficacy across all OI subtypes, injection site reactions, and mild flu-like symptoms [24,26]
Wnt	Monoclonal antibody of SOST (Blosozumab)	Blosozumab (Manufacturer: Eli Lilly and Company, Indianapolis, IN, USA)	Osteoporosis	Subcutaneous injection	Blocking sclerostin, promoting osteoblast activity and bone formation.	Potential risks include long-term safety and efficacy, injection site reactions, and possible cardiovascular events [24,26]
TGF-β/BMP	rhBMP-2	InFuse™ (Manufacturer: Medtronic, Minneapolis, MN, USA)	Spinal fusion and other bone repair procedures	Implantation with biomaterials	Activating the Smad1/5/8 pathway, promoting osteoblast differentiation, bone matrix production, and new bone formation.	Easily degraded by enzymes and rapidly cleared. Inflammatory responses, ectopic bone formation, and cancer risk at the implantation site [22,27,28]
TGF-β/BMP	rhBMP-7	OP-1^®^ (Manufacturer: Stryker Corporation, Kalamazoo, MI, USA)	OI, bone fractures and other bone defects	Local injection or implantation	Activating the Smad1/5/8 pathway stimulates bone formation by activating osteoblast differentiation and bone matrix production.	Swelling, pain, and infection at the implantation site, and the risk of ectopic bone formation [22,27,28]
PTH/PTHrP	PTH (1–34) (Teriparatide)	Forteo^®^ (Manufacturer: Eli Lilly and Company, Indianapolis, IN, USA)	Osteoporosis	Intermittent subcutaneous injection	Stimulating osteoblasts to increase bone formation while also reducing bone resorption.	Hypercalcemia, orthostatic hypotension, dizziness, and an increased risk of osteosarcoma in animal studies, making long-term use a concern [29,30,31]
PTH/PTHrP	PTHrP analog (Abaloparatide)	TYMLOS^®^ (Manufacturer: Radius Health, Inc., Boston, MA, USA)	Osteoporosis	Subcutaneous injection	Activates the parathyroid hormone receptor to stimulate osteoblast activity, thereby promoting bone formation and increasing bone mineral density.	Hypercalcemia, dizziness, headache, and an increased risk of osteosarcoma in animal studies. Long-term use should be limited [29]

Abbreviations: SOST = sclerostin, OI = osteogenesis imperfecta.
